# Diabetes and Coronary Heart Disease: A Risk Factor for the Global Epidemic

**DOI:** 10.1155/2012/697240

**Published:** 2012-10-18

**Authors:** Maguy Chiha, Mario Njeim, Edgar G. Chedrawy

**Affiliations:** ^1^Division of Endocrinology, Department of Medicine, Loyola University Medical Center, 2160 South First Avenue, Fahey Bldg, Maywood, IL 60153, USA; ^2^Division of Cardiovascular Medicine, Department of Medicine, Henry Ford Hospital, 2799 West Grand Boulevard, Detroit, MI 48202, USA; ^3^Cardiovascular and Thoracic Surgery, University of Illinois at Chicago and Vanguard Weiss Memorial Hospital, 4646 North Marine Drive, Chicago, IL 60640, USA

## Abstract

Cardiovascular disease remains a leading cause of death in the United States and the world. In this we will paper focus on type 2 diabetes mellitus as a risk factor for coronary heart disease, review the mechanisms of atherogenesis in diabetics, the impact of hypertension and the treatment goals in diabetics, the guidelines for screening, and review the epidemiologic consequences of diabetes and heart disease on a global scale. The underlying premise to consider diabetes a cardiovascular disease equivalent will be explored as well as the recommendations for screening and cardiac testing for asymptomatic diabetic patients.

## 1. Introduction

Cardiovascular disease is currently responsible for 30% of all deaths worldwide with most of the burden now occurring in developing countries [1]. After a peak around 1968, death from coronary heart disease (CHD) has declined significantly in the United States [2]. Based on a statistical mortality model previously validated in Europe, New Zealand, and China [3–6], Ford et al. estimated that 47% of the decrease in mortality from coronary heart disease in the United States between 1980 and 2000 was attributed to advances in medical therapies including treatment of acute coronary syndromes and heart failure. Approximately 44% of the reduction was secondary to a decline in cardiovascular risk factors including hypercholesterolemia, hypertension, smoking, and physical inactivity. This improvement was partially counterweighted by an increase in the prevalence of diabetes and body mass index [7]. In contrast to the United States, the cardiovascular disease epidemic continues to rapidly evolve on a global level and is currently responsible for twice as many death in developing compared to developed countries [1]. In low- and middle-income countries, cardiovascular risk factors especially smoking and obesity continue to increase in prevalence and affect a larger proportion of younger patients [8]. Cardiovascular mortality has been reported 1.5 to 2 times higher among the working population in India, South Africa, and Brazil compared to the United States [8]. 

Diabetes mellitus is associated with an increased risk of cardiovascular death and a higher incidence of cardiovascular diseases including coronary artery diseases (CAD), congestive heart failure (CHF) [9], and atrial fibrillation [10]. The mechanisms underlying the association between glucose homeostasis and each of myocardial dysfunction and atrial fibrillation remain mostly speculative. In contrast the relationship between abnormal glucose homeostasis and coronary artery disease has been the center of extensive basic science, epidemiological, and therapeutic research studies. 

In this paper we will focus on type 2 diabetes mellitus as a risk factor for CAD, review the mechanisms of atherogenesis in diabetes, the impact of hypertension on the treatment goals in diabetes, the guidelines for CAD screening, and review the epidemiologic consequences of diabetes and heart disease on a global scale.

## 2. Prevalence of Cardiac Disease among Diabetics

Diabetes mellitus has been well described as a cardiovascular risk factor in developed countries. In the Framingham study, the incidence of cardiovascular disease among diabetic men was twice that among nondiabetic men, and similarly was three times more elevated in diabetic women compared to nondiabetic women [11]. In the Copenhagen City heart Study, the relative risk of incident myocardial infarction was 2 to 3 fold increased in diabetics compared to nondiabetics, independent of the presence of other known cardiovascular risk factors (such as hypertension) [12]. In a recent meta-analysis by Berry et al. reviewing the lifetime risks of cardiovascular disease, 18 studies involving 257,384 men and women were reviewed. Patients were stratified by blood pressure, cholesterol level, smoking status, and diabetes status and by age group as well as gender and race. Significant differences were noted in the lifetime risk of cardiovascular disease, with substantially lower risk of fatal and nonfatal cardiovascular disease among participants with no risk factors. The trend was observed in both genders, across all age groups as well as among all races [13]. Furthermore the *differential* impact of diabetes on coronary artery disease mortality in men and women has been the subject of multiple studies; Lee et al. reported the relative risk of coronary heart disease mortality to be 2.5 in women, compared to 1.85 in men [14]. Even modest elevations in blood glucose, without a diagnosis of diabetes, have been linked to increased risk for development of CAD independent of other recognized risk factors when reviewed in a population of predominantly male, nondiabetic veterans [15]. 

### 2.1. Diabetes in the Developing Countries

 Our current knowledge about the epidemiology of diabetes mellitus and its association with cardiovascular disease is mainly derived from studies done in populations of European origin. There is however increasing data available from other ethnic groups and emerging countries suggesting a substantial contribution of diabetes to the worldwide epidemics of cardiovascular disease. King et al. used a global database from the World Health Organization in addition to demographic projections from the United Nations to generate numerical estimates for the prevalence of diabetes in all countries of the world. The authors reported that the prevalence of diabetes mellitus is lower in developing than in developed countries and estimated that it will remain so in 2025. On the other hand, 62% of the diabetic patients worldwide resided in emergent nations in 1995 and this proportion is estimated to reach 75% by 2025 [16]. The INTERHEART study was a case-control study including patients from 52 different countries representing all inhabited continents. It was designed to assess if the association between acute myocardial infarction and various risk factors including diabetes mellitus varies by geographic region, ethnic origin, sex, or age. Diabetes was one of 9 risk factors that were significantly related to acute myocardial infarction in all age groups, genders and regions of the world. After adjustment for all other risk factors, the population attributable risk for diabetes alone was 9.9% in the overall study population. Despite variations across different subpopulations in the prevalence for diabetes, the odds ratio for this risk factor was qualitatively similar in all regions of the world and all ethnic groups [17]. 

## 3. Pathogenesis of CAD in Diabetes

There is a consensus in the literature about an increased prevalence of coronary plaques in diabetic hearts, with such plaques bearing a higher propensity for rupture. In a study by Silva et al., the coronary angiography and angioscopy findings of 55 consecutive patients admitted with unstable angina (31% of which were diabetic) were reviewed. The plaque was ulcerated in 94% of the diabetic patients, versus in only 60% of the nondiabetic patients, and thrombi were found in 94% of diabetic patients versus 55% of nondiabetics [18]. Similarly, in a postmortem study of coronary atherectomy specimens from 47 diabetic and 48 non diabetic patients, Moreno et al. noted a larger lipid content and increased macrophage infiltration and thrombosis in the atheromas of diabetic patients [19]. Multiple mechanisms appear to be involved, including endothelial dysfunction, hypercoagulability, and platelet dysfunction [20], with hyperglycemia being the common trigger.

Hyperglycemia results in multiple biochemical changes, a few of which we will list: an increase in the reduction of nicotinamide adenine dinucleotide (NAD+) to NADH is thought but not proven yet to be a cellular oxidative stressor; an increase in the production of uridine diphosphate (UDP) N-acetyl glucosamine is thought to alter cellular enzymatic function. Very importantly, the glycosylation of proteins in the arterial wall is thought to contribute to diabetic atherosclerosis. The nonenzymatic reaction between glucose and arterial wall proteins results in the formation of advanced glycation end products (AGE), process that is enhanced in hyperglycemia. AGEs are thought to directly interfere with endothelial cell function and accelerate atherosclerosis. Additionally, hyperglycemia increases the formation of reactive oxygen species (ROS); these ROS inhibit endothelial production of nitric oxide, a potent vasodilator and regulator of platelet activation [21]. Furthermore, those ROS prevent the migration of vascular smooth muscle cells into the intimal plaques, a step necessary to the stabilization of coronary plaques. Such plaques then carry an increased risk of rupture, as is known of diabetic coronary plaques [22].

In the event of a plaque rupture, the increased thrombogenesis and platelet dysfunction present in diabetes worsen the clinical consequences of plaque rupture. Circulating glucose molecules freely enter platelets, raising intracellular glucose concentration and leading to activation of protein kinase C, decrease platelet derived NO, and increased expression of glycoprotein Ib (GpIb), a platelet aggregation mediator [20]. This might further explain the enhanced thrombosis in diabetics. In an elegant experimental design, Shechter et al. were able to demonstrate the role of glucose as an independent predictor of platelet dependent thrombosis [23]. Furthermore, insulin has been found to increase serum concentrations of Plasminogen Activator Inhibitor type I (PAI-1) [24] which has been shown to correlate with impaired fibrinolysis [25].

## 4. Hypertension in Diabetes

Hypertension is often found at the time of diagnosis of type 2 diabetes even in the absence of microalbuminuria [26]. It is postulated that hyperinsulinemia, arterial stiffness as well as extracellular fluid volume expansion all play a role. Indeed hyperinsulinemia is linked to weight gain, as well as increased sympathetic activation [27]. Additionally, when hyperglycemia is mild, it results in increased glucose filtration and subsequent reabsorption at the glomerulus, driving sodium reabsorption with it and causing extracellular fluid volume expansion. 

Treatment of hypertension in diabetes is essential to prevent development of renal disease, retinopathy as well as cardiovascular disease. In the UKPDS trial that looked at patients with type 2 diabetes, achieving lower blood pressure with captopril or atenolol versus placebo resulted in a 24% reduction in diabetes-related end points including microvascular disease, diabetes related deaths, stroke, as well as retinopathy. The achieved blood pressure in the treatment arm was 144/82 mm Hg, compared to 154/87 mm Hg; these benefits required continued control of blood pressure. Indeed upon eight years of followup, the greater reduction of blood pressure that was achieved in the treatment arm disappeared and the reduction in clinical end points was not sustained [28]. 

In the ADVANCE trial, the effect of intensive blood pressure control on cardiovascular disease in patients with long standing type 2 diabetes at high risk for vascular disease was studied. A combination of perindopril-indapamide was compared to placebo. After more than four years of treatment, a lower rate of major macrovascular and microvascular events as well as a lower risk of cardiovascular mortality were noted. The mean blood pressure achieved in the treatment arm was 134.5/74 mm Hg, compared to 140/76 mm Hg in the placebo arm [29]. 

Major guidelines therefore recommend a goal blood pressure of less than 130/80 in diabetic patients [30–32]. The benefits of lower blood pressure goal have not been established. In the blood pressure arm of the ACCORD trial, patients with type 2 diabetes and cardiovascular disease (or two additional risk factors for cardiovascular disease) were randomly assigned to either intensive therapy (goal systolic blood pressure less than 120 mm Hg) or standard therapy (goal systolic blood pressure less than 140 mm Hg). The goals were reached with a mean systolic blood pressure of 119.3 mm Hg in the intensive arm and 133.5 mm Hg in the standard arm. After 4.7 years, there was no difference in the annual rate of the primary composite outcome of nonfatal myocardial infarction, nonfatal stroke, or death from cardiovascular causes between groups. There was no difference in the annual all-cause mortality or death from cardiovascular disease. The annual rates of total and nonfatal stroke was significantly lower in the intensive treatment arm, but serious adverse events attributable to antihypertensive drugs occurred more significantly in the intensive group. Therefore there is no recommendation to achieve a systolic blood pressure of less than 120 mm Hg [33]. Similar results were noted in the SANDS trial which looked at progression of atherosclerosis and left ventricular hypertrophy as well as clinical cardiovascular events in American Indian patients with type 2 diabetes treated to a goal blood pressure of either less than 120/75 mm Hg or less than 115/70 mm Hg. Tight blood pressure control resulted in reduction in the progression of atherosclerosis and left ventricular mass index but no decline in clinical cardiovascular outcomes. More adverse events related to antihypertensive drugs were noted in the intensive therapy arm [34].

### 4.1. Diabetes as a Cardiovascular Disease (CVD) Equivalent and a Poor Prognostic Factor in Acute Coronary Syndromes

Most guideline documents recommend treating cardiovascular risk factors in diabetic patients as aggressively as in patients with established coronary artery disease. In 2002, the National Cholesterol Education Program report designated diabetes as a coronary heart disease equivalent. The report explained that a more intensive prevention strategy is justified in diabetic patients because of their high risk of new CAD within 10 years, and because of the high death rate observed in diabetics who experience a MI [35]. In the following section we will briefly highlight and discuss the evidence behind these 2 observations.

Diabetic patients have twice the risk of myocardial infarction (MI) and stroke of that of the general population [36]. In their cross-sectional study, Haffner et al. identified individuals between age 45 and 64 from the Finnish Social Institution's register and compared the outcomes of type 2 diabetic patients with those of nondiabetic control subjects. During a seven-year followup and after adjustment for other cardiovascular risk factors, the risk of myocardial infarction and the mortality from coronary heart disease were similar in diabetics without prior myocardial infarction and nondiabetics with prior myocardial infarction [37]. This observation, derived from a group of middle-aged and older individuals, has been integrated into clinical practice and served as a premise to consider diabetes a CVD equivalent. Nonetheless, age remains an important factor to take into account when assessing the risk of diabetic patients. In a large population-based retrospective cohort study, Booth et al. reported that diabetes seems to account for a risk equivalent to ageing 15 years. However, younger diabetics (age 40 or younger) do not appear to be at high risk of CVD [38]. A number of comprehensive risk assessment tools taking into account patients' age and risk factors profile are recognized by major scientific societies and have been validated in numerous clinical trials. Such tools are available online and include the Framingham risk calculator [39], the UK Prospective Diabetes Study risk engine [40] and the ADA's Diabetes Personal Health Decisions [41]. 

Diabetic patients are also known to have worse outcomes after an acute coronary syndrome when compared with the general population. Diabetes was an independent mortality risk factor in patients receiving thrombolytic therapy for ST elevation MI in both the GUSTO-I [42] and GISSI-2 trials [43]. Insulin treated patients had the worse outcomes in both trials and diabetes carried a higher adverse impact in women compared to men in the GISSI-2 trial. Newly diagnosed diabetes mellitus at time of presentation with an acute MI was also associated with poorer long-term outcomes in the VALIANT trial [44]. Data from a meta-analysis of 19 trials comparing primary PCI and fibrinolysis in ST elevation MI showed that diabetic patients had a higher mortality reduction with PCI but continued to have worse outcomes than nondiabetics [45]. Similar observations from the VALIANT trial [44] and the OASIS registry [46] demonstrated that diabetics presenting with a non ST elevation MI (NSTEMI) or unstable angina had worse long-term outcomes compared to nondiabetics. OASIS showed one more time a more ominous impact of diabetes on female patients compared to their male counterparts. 

### 4.2. Cardiac Testing in Asymptomatic Patients with Diabetes Mellitus

In addition to the higher incidence of clinically significant cardiovascular events, type 2 diabetes is also associated with a higher rate of subclinical CAD. Non-hemodynamically significant coronary lesions can remain latent before resulting in myocardial ischemia. More importantly diabetic autonomic neuropathy can impair ischemia awareness and has been associated with an increased risk of cardiovascular mortality [47]. Noninvasive computed tomography (using coronary artery calcium (CAC) scoring or angiography) is now capable of detecting asymptomatic CAD even before the onset of silent ischemic electrocardiographic changes and coronary perfusion defects during stress testing. In a prospective study of 510 asymptomatic patients with uncomplicated type 2 diabetes, significant CAC (a reliable marker of atherosclerosis) was seen in 46.3% of the patients. The extent of CAC was a strong predictor of silent ischemia by radionuclide myocardial perfusion imaging and short-term cardiovascular events [48]. The DIAD trial randomized 1123 asymptomatic type 2 diabetics to adenosine stress radionuclide myocardial perfusion imaging or no screening. Stress imaging identified silent ischemia in 22% of the screened patients [49]. A retrospective observational study of 1899 asymptomatic patients with type 2 diabetes showed that stratifying the patients according to the number of additional CVD risk factors they have did not affect their likelihood of having an abnormal myocardial perfusion test and significant coronary artery disease. However patients with 2 or more additional risk factors had a higher likelihood of having more severe CAD with unfavorable angiographic anatomy not amenable to complete percutaneous or surgical revascularization [50]. 

Based on the above findings, clinicians might feel compelled to screen asymptomatic diabetics in an attempt to detect early stages of CAD and implement appropriate therapies. Such an enthusiasm should be tempered by the fact that intensive medical therapy is indicated for all diabetic patients at high risk for CVD which makes screening results unlikely to change management. In addition diagnostic testing can be expensive and can potentially lead to unnecessary procedures and complications. Furthermore the hypothesis that asymptomatic diabetic patients benefit from revascularization remains unproven. The BARI 2D trial randomized 2368 patients with both type 2 diabetes and stable CAD (defined as ≥50% stenosis of a major epicardial coronary artery associated with a positive stess test or ≥70% stenosis of a major epicardial coronary artery and classic angina) to receive prompt revascularization with intensive medical therapy or intensive medical therapy alone. There was no significant difference in death or serious adverse cardiovascular events between both groups [51]. Additional data supporting the futility of screening asymptomatic patients came from the DIAD trial where no significant difference in cardiac death or nonfatal MI was seen between the screening and no-screening groups at a mean followup of 4.8 years [52]. 

Major society guidelines have made the following recommendations about screening for asymptomatic CAD in diabetic patients.

- The 2002 ACC/AHA guidelines update for exercise testing stated that asymptomatic diabetic patients have an increased likelihood of CVD if they have at least one of the following factors: age older than 35, type 2 diabetes greater than 10 years' duration, any additional atherosclerotic risk factor for CAD, presence of microvascular disease, peripheral vascular disease or autonomic neuropathy. The guidelines recommend exercise testing if an individual meeting the above criteria is planning to begin a moderate- to high-intensity exercise (class IIa; level of evidence: C) [53].
- The 2010 ACCF/AHA guidelines for assessment of cardiovascular risk in asymptomatic adults give a class IIa recommendation for measurement of CAC for cardiovascular risk assessment in asymptomatic adults with diabetes who are 40 years of age and older (level of evidence: B). The authors acknowledge that there is no evidence supporting that this imaging test is useful in motivating patients to better adhere to primary prevention measures. The same guidelines give a weak class IIb (level of evidence: C) recommendation to consider stress MPI for advanced cardiovascular risk assessment in asymptomatic adults with diabetes or in patients who have a CAC score of 400 or greater [54].
- In a recent 2012 position statement on standards of medical care in diabetes, the American Diabetes Association (ADA) does not recommend screening for CAD in asymptomatic patients because it does not improve outcomes as long as CVD risk factors are treated (level of evidence: A). The guidelines acknowledge that newer noninvasive CT modalities can identify asymptomatic diabetic patients with a higher CAD burden and a higher risk of future cardiac events. However they consider that the role of these tests beyond risk stratification is unclear with a controversial balance of benefit, cost, and risks [55].

### 4.3. Epidemiologic Consequences of Diabetes and Heart Disease on a Global Scale

The number of people with diabetes is increasing due to population growth, aging, urbanization, and increasing prevalence of obesity and physical inactivity [16, 56, 57]. Quantifying the prevalence of diabetes and the number of people affected by diabetes, now and in the future, is important to allow rational planning and allocation of resources. The prevalence of diabetes for all age-groups worldwide was found to be 2.8% in 2000, 6.4% in 2010, and (estimated to be) 7.7% in 2030! The total number of people with diabetes is projected to rise from 171 million in 2000, to 285 million in 2010 to 440 million in 2030 [58]. The prevalence of diabetes is higher in men than women, but there are more women with diabetes than men. The urban population in developing countries is projected to double between 2000 and 2030. Between 2010 and 2030, there will be a 69% increase in numbers of adults with diabetes in developing countries and a 20% increase in developed countries [58]. The public health epidemic of diabetes will certainly affect the growth of these emerging economies. As the prevalence of diabetes increases so will the need for healthcare services (primary, secondary, and tertiary) in these developing countries. Unfortunately, data about the use of effective secondary prevention medications in patients with known cardiovascular disease reflects the importance of this challenge. The Prospective Urban Rural Epidemiological (PURE) study which enrolled between 2003 and 2009 patients with cardiovascular disease from 17 countries with variable levels of income showed that the use of appropriate drugs (antiplatelet drugs, ACE inhibitors or ARBs, or statins) was universally low and decreased in line with reduction of country economic level. The percentage of patients receiving no drugs was 11.2% in high-income countries, 45.1% in upper middle-income countries, 69.3% in lower middle-income countries, and 80.2% in low-income countries [59]. Moreover access to cardiac revascularization including cardiac surgery and catheter-based techniques remains disproportionate in different parts of the world even within Europe and North America [60]. This has led major scientific societies to develop appropriateness and necessity criteria that can guide decision-making and identify the overuse and underuse of revascularization procedures. Historically, coronary artery bypass grafting (CABG) is the established method of revascularization in patients with diabetes and multivessel coronary disease, but with advances in percutaneous coronary intervention (PCI), there is uncertainty whether CABG remains the preferred method of revascularization. Kapur et al. studied a total of 510 diabetic patients with multivessel or complex single-vessel coronary disease from 24 centers and randomized the patients to PCI plus stenting (and routine abciximab) or CABG [61]. The primary outcome was a composite of all-cause mortality, myocardial infarction (MI), and stroke, and the main secondary outcome included the addition of repeat revascularization to the primary outcome events. At 1 year of followup, the composite rate of death, MI, and stroke was 10.5% in the CABG group and 13.0% in the PCI group (hazard ratio (HR): 1.25, 95% confidence interval (CI): 0.75 to 2.09; *P* = 0.39), all-cause mortality rates were 3.2% and 3.2%, and the rates of death, MI, stroke, or repeat revascularization were 11.3% and 19.3% (HR: 1.77, 95% CI: 1.11 to 2.82; *P* = 0.02), respectively [46]. Regardless of the revascularization approach (PCI versus CABG), the overall increase in prevalence will lead to an increased demand of (tertiary) services for patients who suffer from diabetes and its most fatal complication (cardiovascular disease). The result will be an increased burden on the emerging economies of these developing countries.

## 5. Conclusion

Diabetes mellitus is associated with an increased risk of cardiovascular death and a higher incidence of cardiovascular diseases including coronary artery disease. The substantial rise in prevalence of diabetes will ultimately lead to a huge increase in the demand for primary, secondary, and tertiary healthcare services globally. The need for appropriate screening and cardiac testing is crucial to help better manage the end result (cardiovascular disease) of this global epidemic.
